# The effects of HIF-1alpha on gene expression profiles of NCI-H446 human small cell lung cancer cells

**DOI:** 10.1186/1756-9966-28-150

**Published:** 2009-12-10

**Authors:** Jun Wan, Jinben Ma, Ju Mei, Genfa Shan

**Affiliations:** 1 Department of Cardiothoracic Surgery, Xinhua Hospital, Shanghai Jiaotong University School of Medicine, Shanghai 200092, PR China

## Abstract

**Background:**

Gene targeted therapy refers to any therapy focused on one of the many biological features of the tumor. Such features are mediated by specific genes that are involved in tumor metastasis, recurrence, poor response to chemotherapy and others. Hypoxia is an important pathognomonic feature of many malignant tumors including SCLC (small cell lung cancer). HIF-1alpha, which is induced by hypoxia, is the most important regulatory factor of many specific genes that can influence the biological features of tumors.

**Methods:**

In this study, we tried to elucidate the changes in gene expression profiles of SCLC NCI-H446 cells mediated by HIF-1alpha. According to different treatments of cells, three experimental pairwise comparisons were designed: hypoxia group vs. control group, Ad5-HIF-1alpha group vs. Ad5 group, and Ad5-siHIF-1 alpha group Vs Ad5 group.

**Results:**

Results from the analysis of gene expression profiles indicated that there were 65 genes upregulated and 28 genes downregulated more than two-fold in all three experimental pairwise comparisons. These genes were involved in transport, signal-transduction, cell adhesion/motility, growth factor/cytokines, transcription, inflammatory response, metabolic process, in addition to others. SOCS1, IGFBP5, IL-6 and STAT3 were also upregulated at protein level. SOCS1 could significantly induce apoptosis and suppress growth of NCI-H446 cells but HIF-1alpha could induce growth and suppress apoptosis.

**Conclusions:**

Through this research, we are trying to find novel functional genes that are mediated by HIF-1alpha and provide the theoretical basis for new therapeutic targets. HIF-1 alpha maybe upregulate the expression of SOCS1 through mediation of STAT3 and IL-6. In addition, SOCS1 could significantly induce apoptosis and suppress growth of NCI-H446 cells. This was contrary to HIF-1alpha and it indicated that there might be an antagonism effect between HIF-1alpha and SOCS1 on regulating growth and apoptosis of NCI-H446 cells.

## Background

Lung cancers used to be categorized into small cell lung cancer (SCLC) and non-small cell lung cancer (NSCLC). About 13%-15% of all lung cancers worldwide are SCLC. As a type of malignant tumor, SCLC shows many general clinical manifestations such as early metastasis, frequent recurrence and tendency toward poor response to chemotherapy. Some authors [1,2] have previously demonstrated that these clinical manifestations are partially the result of a series of biological changes that occurred in tumor cells when responding to hypoxia. In other words, the hypoxic microenvironment promotes the malignant development of the tumor. In this biochemical process, the hypoxia-inducible factor-1alpha subunits (HIF-1alpha) play an important role. Expression of HIF-1alpha has been confirmed to be unregulated in hypoxic conditions and degraded in normoxic conditions [3,4]. Changes in gene expression directly or indirectly induced by HIF-1alpha have extended to over 100 genes to date. Through mediating the expression of some relevant functional genes, HIF-1alpha influences the pathways of metabolic adaptation, erythropoiesis, angiogenesis and vascular tone, cell growth and differentiation, survival and apoptosis, and is therefore a critical factor in many biological features of the majority of solid tumors [5], including SCLC. It has been verified that multiple genes and their functions are involved in the occurrence and development of SCLC [6]. However, one question that remains to be answered is how the hypoxic microenvironment changes the gene expression profile of SCLC cells by the regulational activation of HIF-1alpha. To imitate the hypoxic microenvironment of the tumor in vivo, we cultured SCLC NCI-H446 cells in a hypoxic incubator. Additionally, we modified SCLC NCI-H446 cells with Ad5-HIF-1alpha (an adenovirus encoding a active form of HIF-1alpha that is resistant to O2-dependent degradation) and incubated these cells in a normoxic incubator. In order to further clarify the effect on the gene expression profile of NCI-H446 cells by HIF-1alpha, we blocked the expression of HIF-1alpha using Ad5-siHIF-1alpha transfection. For these studies, microarray technology was used, which provides a unique opportunity to study the global gene expression profile by providing a molecular portrait of cellular events in a single experiment [7]. In our study, the Human Genome U133A Array approach was utilized to evaluate the changes of the gene expression profile in NCI-H446 cells after culturing in a hypoxic environment and transfection with Ad5-HIF-1alpha or Ad5-siHIF-1alpha. We applied this approach to analyze the differential expression of functional genes. Our investigation tried to identify more novel functional genes that respond to the HIF-1 alpha changes mediated by hypoxia and hoped to provide the theoretical basis for gene targeted therapy in SCLC. In addition, we found that SOCS1 (which can negatively regulate growth factor signaling and affect the process of proliferation and apoptosis) was upregulated by HIF-1alpha. There may be an antagonism effect between HIF-1alpha and SOCS1 on inducing growth or suppressing apoptosis of NCI-H446 cells. In our study, we carried out research to address this point.

## Materials and methods

### Cell culture

The NCI-H446 cell line was obtained from the American Type Culture Collection (CAS Shanghai Institutes for Biological Sciences cell bank) and cultured in RPMI-1640 medium (Sigma-Aldrich Co, St. Louis, MO, USA) supplemented with 10% fetal bovine serum (FBS, Hyclone) and 100 μg/ml kanamycin at 37°C in humidified atmosphere containing 5% CO2 and 20% O2. The medium was routinely changed 2-3 d after seeding. All experiments were performed using exponentially growing cells and repeated at least 3 times.

### Adenoviral construction and cell transfection

We used Ad5 (full name: tumor-specific replication-defective adenovirus type 5) as the vector. Ad5- HIF-1alpha, Ad5-siHIF-1alpha, Ad5-SOCS1 and Ad5-siSOCS1 were constructed and gifted from the Viral-Gene Therapy department of Shanghai Eastern Hepatobiliary Surgery Hospital. The cells in the microarray analysis were divided into five groups: control group (cells cultured in a normoxic environment with 20% O2), hypoxia group (cells cultured under a hypoxic environment with 1% O2), Ad5 group (cells transfected with Ad5), Ad5-HIF-1alpha group (cells transfected with Ad5-HIF-1alpha) and Ad5-siHIF-1alpha group (cells transfected with Ad5-siHIF-1alpha and cultured under hypoxic environment with 1% O2). For transfection, cells were cultured in 6-well plates and exposed to viral supernatant in the absence of cytokines and serum with different multiplicities of infection (MOIs): the number of plaque-forming units (pfu) per cell. The efficiency of transfection was estimated by determining the percentage of enhanced green fluorescence protein (EGFP)-positive cells in cells infected with Ad5-EGFP. To establish optimal conditions for NCI-H446 cells by adenoviral gene transfer, different titers of Ad5-EGFP were used. After transfection for 3 days, half of the virus-containing medium was replaced for the first time, and then plates were further incubated and all the medium was changed every 2 days. According to a report by Meng Jiang [8], we imitated the hypoxic microenvironment in vivo by putting the cells into a hypoxic chamber with an auto purge airlock (Thermo Forma, Tri-tube, USA). Environmental hypoxic conditions were established in an airtight humidified chamber that was continuously flushed with a gas mixture containing 1% O2, 5% CO2 and 94% N2 at 37°C.

### RNA extraction, Microarray hybridization and data analysis

All the cells were washed gently with ice-cold phosphate-buffered saline (PBS) and lysed with 3 ml Trizol (Invitrogen, San Diego, CA, USA). According to the manufacturer's protocol, total RNA was extracted and purified with the RNAeasy kit (Qiagen, USA). The concentration of total RNA was measured by a Biophotometer (Eppendorf, Germany), and the quality of purified RNA was confirmed by agarose gel electrophoresis using ethidium bromide staining. cDNA was synthesized from each RNA sample using SuperScript System (Invitrogen) as a template for the preparation of biotin-labeled cRNA according to the GeneChip IVT Labeling Kit. The hybridization fluid was prepared and Biotin-labeled cRNA was hybridized to the GeneChip Human Genome U133 Plus 2.0, washed, stained with phycoerythrin-streptavidin and scanned according to the manufacturer's protocol. The microarray contained 54,614 human gene probe sets, each of which consisted of 11 probe pairs corresponding to a single mRNA transcript. After being saved as raw image files, all the data were converted into probe sets and analyzed by the GCOS software based on the method of normalization. Annotation by Unigene database http://www.ncbi.nlm.nih.gov/entrez/query.fcgi?db_unigene, gene number, gene symbol, and gene description were carried out using the database http://david.abcc.ncifcrf.gov/summary.jsp and Affymetrix databases. The results are presented as the ratios of the hypoxia group vs. control (normoxia) group, Ad5-HIF-1alpha group vs. Ad5 group1 and Ad5-si HIF-1alpha group vs. Ad5 group2. Ratio values with an increase or decrease of more than 2 folds were defined as differential expression. The primary data sets are all available at http://www.hopkins-genomics.org/expression.html.

### Selecting genes for real-time quantitative PCR

The microarray data were verified by real-time quantitative PCR. Six upregulated genes were selected to validate and PCR primer pairs were as follows:

human IGFBP5: sense 5'-TGCCCAGAAAATGAAAAAGG-3'and

antisense 5'-GGATGACACAGCGTGAGAGA -3'

human IRS4: sense 5'-TACGGCAATGGCTTTATCAC-3' and

antisense 5'-CCCTCCTGCAACTTCTCAAT-3'

human TNFAIP6: sense 5'-TTTCAAGGGTGCCAGTTTCG-3' and

antisense 5'-GGGAGGCCAGCATCGTGTA-3'

human SOCS1: sense 5'-TAGCACACAACCAGGTGGCA-3'and

antisense 5'-GCTCTGCTGCTGTGGAGACTG-3'

human IL-6: sense 5'-CGGGAACGAAAGAGAAGCTCTA-3' and

antisense 5'- CGCTTGTGGAGAAGGAGTTCA-3'

human VEGF-A: sense 5'- CCATGAACTTTCTGCTGTCTT-3' and

antisense 5'-TCGATCGTTCTGTATCAGTCT-3'

Five downregulated genes were selected to validate and PCR primer pairs were as follows:

Human IGFBP3: sense 5'-GACGTATCTAGCAGCTGTCT-3'and

antisense 5'- CGAGGTCTCATGATCTCTCT -3'

Human ZNF569: sense 5'-GGAAAGAAACGACTGGGAGC-3' and

antisense 5'-CGACTAGACGCTATTGTGATT-3'

Human SOCS-2: sense 5'-CCTTTATCTGACCAAACCGCTCTA-3'and

antisense 5'-TGTTAATGGTGAGCCTACAGAGATG-3'

Human SIRPa: sense 5'-GGCGGGTGAGGAGGAGCTGCAGGTGAT-3' and

antisense 5'-GCGGGCTGCGGGCTGGTCTGAATG-3'

Human XRCC4: sense 5'-AAGATGTCTCATTCAGACTTG-3'and

antisense 5'-CCGCTTATAAAGATCAGTCTC-3'

Real-time PCR was performed using SYBR ExScript RT-PCR Kit according to the manufacturer's protocol (Takara Biotechnology (Dalian) Co. Ltd., Dalian, China) and using the iCycler Real-Time PCR Detection System (BioRad). All the RNA samples, which were chosen from the microarray samples, were run in duplicate on 96-well optical PCR plates. The thermal cycling conditions were as follows: 1 cycle of 95.0°C for 10 min; 40 cycles of 95.0°C for 5 s; 60.0°C for 30 s; and 81 cycles of 55.0°C for 10 min (with an increase set point temperature after cycle 2 by 0.5°C). GAPDH was used as an internal control. The primers used for SYBR Green real-time PCR were designed according to the NCBI website http://www.ncbi.nlm.nih.gov and were synthesized by Shanghai Sangon Biological Engineering Technology & Services Co., Ltd. The relative changes in gene expression were calculated using the equation: relative changes in gene expression = 2^**-ΔΔCT **^where ΔCt = Ct_target_- Ct_GAPDH _and ΔΔCt = ΔCt_Ad5-HIF-1alpha_-ΔCt_Ad5_, ΔΔCt = ΔCt_Ad5_-ΔCt_Ad5-siHIF-1a _or ΔΔCt = ΔCt_Hypoxia_-ΔCt_normoxia_

### Western blot analysis

The cells of each group were washed with mild PBS to remove the remaining medium, and cellular proteins were extracted by disrupting cells in RIPA lysis buffer (Beyotime Institute of Biotechnology): 50 mM Tris pH 7.4, 1 mM EDTA, 250 mM NaCl, 0.1% NP40, 1% Triton X100, 0.5% SDS, 0.25% DOC, 1 mM NaF, 5 mM NaVO3, 1 mg/ml aprotinin, 1 mg/ml leupeptin, 1 mg/ml pepstatin, and 1 mM PMSF. The protein was electrophoresed on 12% SDS-polyacrylamide gels and transferred to a PVDF membrane. The membranes were then blocked at room temperature for 1 h with 5% non-fat milk in Tris buffered saline containing Tween20 (TBST). The rabbit anti-human primary antibodies (Wuhan Boster Biological Engineering Technology Limited Company) that detect IGFBP5, SOCS1, IL-6 and STAT3(Signal Transducer and Activator of Transcription 3)were incubated with membranes overnight at 4°C. The membranes were subsequently incubated with goat anti-rabbit peroxidase-conjugated secondary antibodies, and immunoreactivity was detected by using an enhanced Chemiluminescence kit and captured on X-ray film. β-actin was used as an internal control.

### Analysis of the effect on cell growth and apoptosis by HIF-1alpha and SOCS1

In this study, all cells were divided into 7 groups: Ad5 group - transfection with Ad5 (control group); Ad5-HIF-1a group - transfection with Ad5-HIF-1 alpha; Ad5-si HIF-1alpha group - transfection with Ad5-siHIF-1alpha; Ad5-SOCS1 group - transfection with Ad5-SOCS1; Ad5-siSOCS1 group - transfection with Ad5-siSOCS1; Ad5-HIF-1alpha/siSOCS1 group - co-transfection with Ad5-HIF-1alpha and Ad5- siSOCS1; Ad5-siHIF-1alpha/SOCS1 group - co-transfection with Ad5-siHIF-1 alpha and Ad5-SOCS1; Ad5-HIF-1alpha/SOCS1 group - co-transfection with Ad5-HIF-1 alpha and Ad5-SOCS1. NCI-H446 cells of each group were prepared as a cell suspension and plated at a density of 1 × 10^4 ^cells/well into 6-well plates. Every 24 h, 3 wells were trypsinized for cell counting and repeatedly counted for 7 d to draw the growth curve. Then, cells of each group were washed with PBS and fixed in 70% ethanol for 24 h at 4°C. The fixed cells were resuspended in PBS. After incubation for 10 min, the apoptotic rates were analyzed by terminal transferase dUTP nick-end labeling (tunel stain)and all the procedures were performed according to tunel kit's protocol(Beyotime Institute of Biotechnology). After DAB coloration we began to calculate the apoptosis rate by using the formula: apoptosis rate = number of tunel positive cells/number of total cells.

### Statistical analysis

All experiments were carried out in triplicate. Student's t test or ANOVA was used to compare parameters between the different study groups. A P value of less than 0.05 was considered statistically significant. The statistical analyses were performed with the Windows SPSS 13.0 package.

## Results

### Transfection efficiency and expression of HIF-1alpha

After adenoviral construction was completed, they were used to infect NCI-H446 cells. Using fluorescent microscopy, we observed that the transfection efficiency of the adenoviral vectors into cells was high and reached more than 95% at an MOI of 50. We selected this group to detect the mRNA expression of HIF-1alpha at different stages by real-time quantitative PCR. The primer pairs were: human HIF-1alpha: sense 5'-CAT CAG CTA TTT GCG TGT GAG GA-3' and antisense 5'-AGC AAT TCA TCT GTG CTT TCA TGT C-3'. Results show that 60 h after transfection, the expression of HIF-1alpha mRNA reach the highest level in the Ad5- HIF-1alpha group and the lowest level in the Ad5-siHIF-1alpha group. Therefore, for the following studies human NCI-H446 cells were transduced with Ad5, Ad5- HIF-1alpha or Ad-siHIF-1alpha for 60 h at an MOI of 50.

### Microarray analysis of the gene expression profile of human small cell lung cancer NCI-H446 cells in response to hypoxia by HIF-1alpha

**Figure 1:**
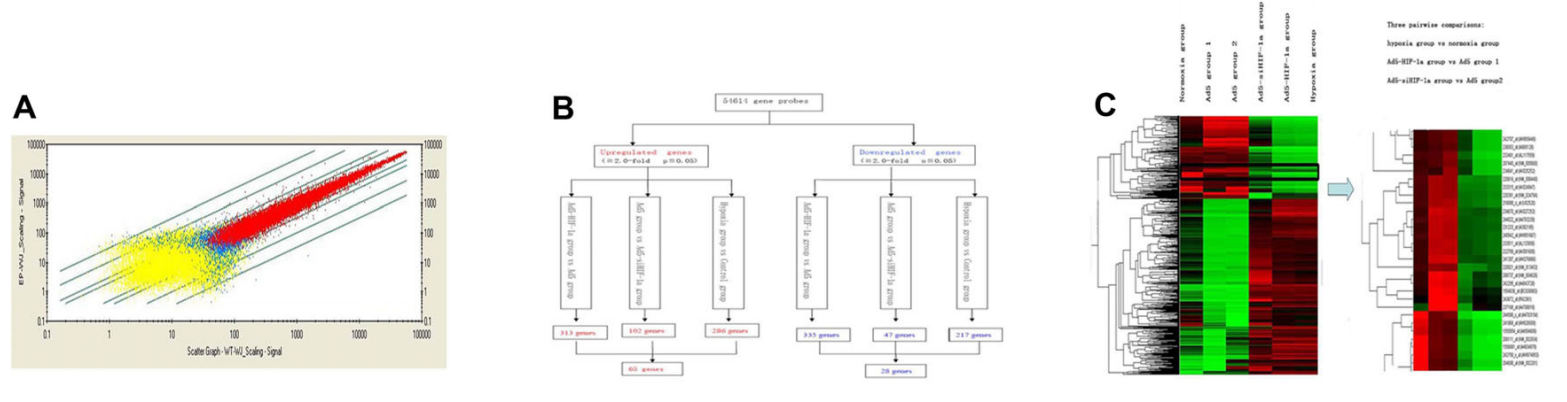
**Microarray and data analysis (A) Scatter graph of gene chip scanning signals: Scatter plot of the normalized microarray datasets resulting from analysis of human SCLC NCI-H446 cells**. All 54,614 gene probes are represented in this plot. **(B) **Experimental design and summary of results: Text in red indicates the total number of genes upregulated in 3 experimental conditions (Ad5-HIF-1alpha vs. Ad5; Ad5 vs. Ad5-siHIF-1alpha; hypoxia vs. control-normoxia). Text in blue indicates the total number of genes downregulated in 3 experimental conditions (same as above). Finally, 65 genes were upregulated and 28 genes were downregulated in all 3 pairwise comparisons. **(C) **Hierarchical clustering analysis of gene expression profiles of three pairwise comparisons (Ad5-siHIF-1alpha group vs. Ad5 group1, Ad5-HIF-1alpha group vs. Ad5 group2, hypoxia group vs. normoxia group). The normalization of all the data of genes with differential expression was handled by clustering analysis using software Gene Spring 7.0. The graph of clustering analysis on the right side is the magnification about the local region (as marked by black border) of the total clustering analysis.

To evaluate the effect of HIF-1alpha on gene expression profiles, cells from all 5 groups were harvested for isolation of total RNA, which was used to synthesize cDNA and labeled cRNA for hybridization to microarrays containing 54,614 gene probes. The experimental protocol was independently performed 3 times. We used the comparative analysis algorithm provided by Genespring to compare differences between the hypoxia group and control group, Ad5-HIF-1alpha group and Ad5 group, Ad5-siHIF-1alpha group and Ad5 group. The genes regulated by HIF-1alpha were determined using a 2.0-fold change cutoff value because this cutoff captured many, but not all of the genes that were previously identified as target genes of HIF-1alpha. We identified 65 gene probes with increased expression (more than 2.0-fold) in the hypoxia and Ad5-HIF-1alpha groups but decreased expression (more than 2.0-fold) in the Ad5-siHIF-1alpha group; 28 gene probes were identified with decreased expression (more than 2.0-fold) in the hypoxia and Ad5-HIF-1alpha groups but increased expression (more than 2.0-fold) in the Ad5-siHIF-1alpha group (Figure 1B). As supplements for the above-mentioned analysis, we performed scatter-graphs of gene chip scanning signals (Figure 1A) and the clustering analysis of gene expression (Figure 1C) to describe the differential expression in response to HIF-1alpha.

### Major functional categories of upregulated genes in response to hypoxia by HIF-1alpha

**Figure 2:**
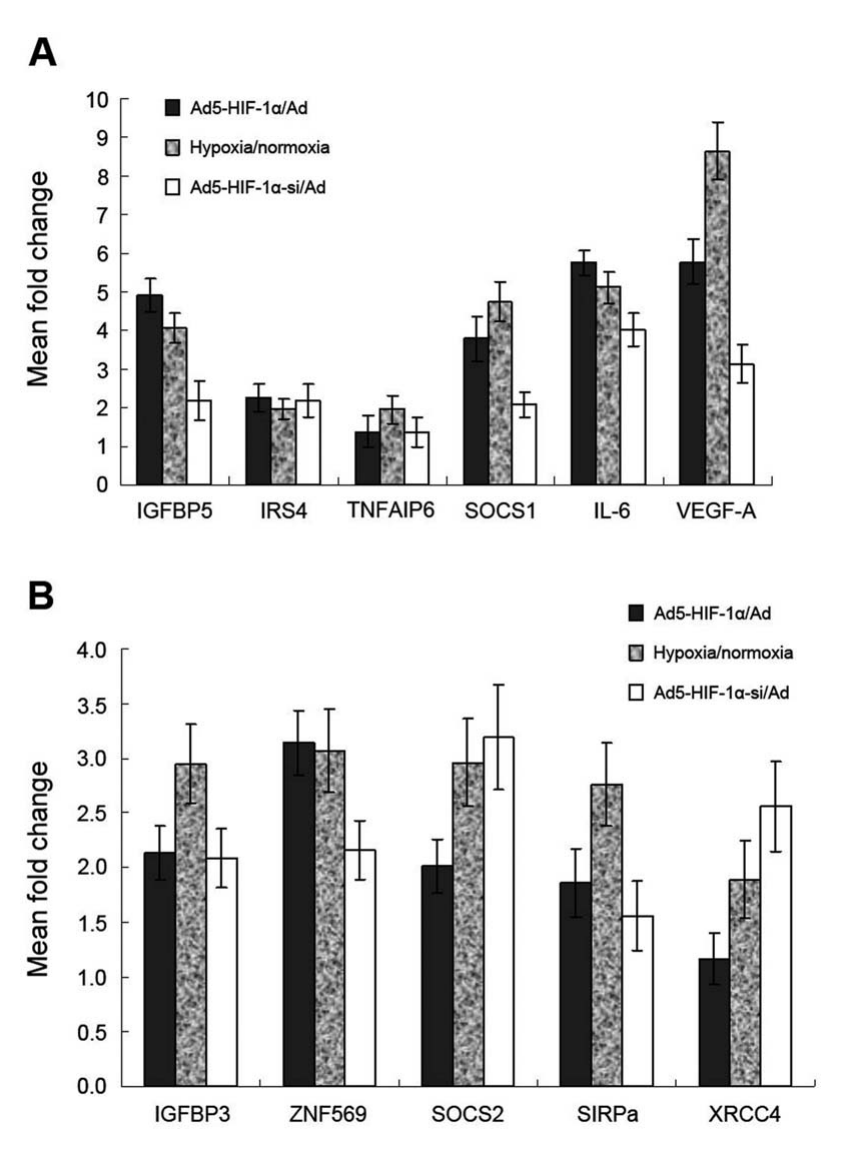
**Real-time PCR analysis of upregulated or downregulated gene expression in response to HIF-1alpha (A) Aliquots of the same RNA preparations used for microarray hybridization were analyzed by quantitative real-time PCR**. In three pairwise comparisons, the upregulation-folds of IGFBP5, IRS4, TNFAIP6, SOCS1, IL-6, VEGF-A mRNA expression were calculated. The mean and standard error are shown (p < 0.05). **(B) **Aliquots of the same RNA preparations used for microarray hybridization were analyzed by quantitative real-time PCR. In three pairwise comparisons, the downregulation-folds of IGFBP3, ZNF569, SOCS2, SIRPa and XRCC4 mRNA were calculated. The mean and standard error are shown (p < 0.05).

Analysis of genes that were upregulated revealed several large categories of gene products associated with immune response, transport, signal transduction, cell adhesion/motility, growth factor/cytokines, transcription, inflammatory response, metabolic process, apoptosis and others (Table 1). The gene most highly upregulated by HIF-1alpha was CLIC2. The largest groups upregulated by HIF-1alpha in NCI-H446 cell were genes associated with transport and the metabolic process. Among the genes associated with transport, the largest category was the SLC (solute carrier) gene family including SLC6A2, SLC9A2, SLC38A6, SLC16A6, SLC41A2, SLC12A8, SLC12A6, SLC39A8 and SLCO4A1. The genes of the SLC family such as SLC2A14 and SLC2A3 and the AKR1 (aldo-keto reductase 1) family such as AKR1C1, AKR1C2, AKR1C3 and AKR1B10 were associated with the metabolic processes of tumor cells. Ten genes were identified that encode cytokines and growth factors including the known target genes of HIF-1alpha such as VEGF, IGFBP5, PDGFC and CRLF1. Novel upregulated genes that might be implicated as target genes of HIF-1alpha including TNFAIP6, HMOX1, HMGA2, HEY1, PLA2G4A and SOCS1. Another large category of target genes encoded transcription factors; among these CREM and ZNF277 were target genes of HIF-1alpha. Among the genes encoding inflammatory response factors, 8 genes (TNFAIP6, IL1R1, BDKRB1, C4A, PTGS2, TNFRSF11B, FN1 and IL6) were upregulated. No gene encoding for inflammatory response factors were downregulated by HIF-1alpha. To validate the microarray data, aliquots from the same RNA preparations were analyzed by quantitative real-time PCR for six genes: IGFBP5, IRS4, TNFAIP6, SOCS1, IL-6, VEGF-A. The results of the real-time PCR showed a similar trend of regulation as the microarray data despite the different upregulational fold (Figure 2A).

**Table 1:** 65 genes upregulated by HIF-1alpha more than 2.0-fold in three pairwise comparisons

UniGeneID	Gene name	Gene Symbol	Fold change(ratio ≥ 2)		
			
			Ad5-HIF-1alpha/Ad5	Ad5-siHIF-1alpha/Ad5	Hypoxia/normoxia
**Immune response**					
Hs.351812	C-type lectin domain family 4, member C	CLEC4C	12.99	-9.66	17.54
Hs.190622	DEAD (Asp-Glu-Ala-Asp) box polypeptide 58	DDX58	5.28	-3.12	4.77
Hs.163173	interferon induced with helicase C domain 1	IFIH1	3.73	-2.07	4.15
Hs.529053	complement component 3	C3	2.29	-2.10	3.17
**Transport**					
Hs.655445	chloride intracellular channel 2	CLIC2	12.12	-3.92	10.46
Hs.257352	apolipoprotein L, 6	APOL6	8.57	-3.98	7.92
Hs.78036	solute carrier family 6, member 2 (neurotransmitter transporter, noradrenalin)	SLC6A2	5.28	-4.01	4.79
Hs.250083	solute carrier family 9, member 2 (sodium/hydrogen exchanger)	SLC9A2	2.64	-2.19	3.17
Hs.200738	solute carrier family 38, member 6	SLC38A6	2.46	-2.94	2.86
Hs.42645	solute carrier family 16, member 6 (monocarboxylic acid transporter 7)	SLC16A6	2.30	-3.16	4.90
Hs.577463	solute carrier family 41, member 2	SLC41A2	2.32	-4.23	5.19
Hs.658514	solute carrier family 12, member 8 (potassium/chloride transporters)	SLC12A8	2.14	-3.01	3.75
Hs.510939	solute carrier family 12, member 6	SLC12A6	2.12	-2.01	3.41
Hs.288034	solute carrier family 39, member 8 (zinc transporter)	SLC39A8	2.05	-2.92	4.35
Hs.235782	solute carrier organic anion transporter family member 4A1	SLCO4A1	2.05	-2.88	5.12
**Signal transduction**					
Hs.592215	insulin receptor substrate 4	IRS4	6.96	-5.79	5.13
Hs.20961	G protein-coupled estrogen receptor 1	GPER1	6.50	-3.05	7.99
Hs.75199	protein phosphatase 2, regulatory subunit B beta isoform	PPP2R5B	3.48	-2.51	6.70
Hs.145404	phosphatidylinositol-specific phospholipase C X domain containing 3	PLCXD3	3.40	-4.91	3.46
Hs.497402	leucine-rich repeat-containing G protein-coupled receptor 6	LGR6	2.46	-2.19	4.10
Hs.458414	interferon induced transmembrane protein 1	IFITM1	2.29	-3.43	2.86
Hs.645475	amphiregulin (schwannoma-derived growth factor)	AREG	2.05	-3.07	3.86
**Cell adhesion/motility**					
Hs.143250	tenascin C (hexabrachion)	TNC	5.28	-3.23	6.44
Hs.2375	egf-like module containing, mucin-like, hormone receptor-like 1	EMR1	3.48	-3.31	4.57
Hs.415762	lymphocyte antigen 6 complex, locus D	LY6D	2.30	-4.30	3.61
Hs.479439	protocadherin 7	PCDH7	2.00	-3.29	2.70
Hs.2962	S100 calcium binding protein P	S100P	4.59	-2.16	4.32
Hs.332012	immunoglobulin superfamily, member 8	IGSF8	2.00	-2.47	2.85
**Growth factors/cytokines**					
Hs.73793	Vascular endothelial growth factor-A	VEGF-A	6.76	-3.98	15.40
Hs.437322	tumor necrosis factor, alpha-induced protein 6	TNFAIP6	6.96	-4.75	12.17
Hs.635441	insulin-like growth factor binding protein 5	IGFBP5	4.83	-4.45	9.80
Hs.517581	heme oxygenase (decycling) 1	HMOX1	2.64	-2.73	4.58
Hs.505924	high mobility group AT-hook 2	HMGA2	2.63	-2.83	2.09
Hs.234434	hairy/enhancer-of-split related with YRPW motif1	HEY1	2.60	-2.15	3.89
Hs.570855	platelet derived growth factor C	PDGFC	2.26	-3.21	4.37
Hs.497200	phospholipase A2, group IVA	PLA2G4A	2.14	-2.55	6.67
Hs.114948	cytokine receptor-like factor 1	CRLF1	2.00	-3.05	4.75
Hs.50640	suppressor of cytokine signaling 1	SOCS1	7.46	-7.13	8.06
**Transcription**					
Hs.501778	tripartite motif-containing 22	TRIM22	4.56	-4.14	4.47
Hs.1706	interferon regulatory factor 9	IRF9	3.73	-2.16	3.90
Hs.567641	myocardin	MYOCD	3.03	-2.08	3.58
Hs.655904	zinc finger protein 277	ZNF277	2.13	-2.74	2.37
Hs.200250	cAMP responsive element modulator	CREM	2.05	-2.31	3.45
**Inflammatory response**					
Hs.437322	tumor necrosis factor, alpha-induced protein 6	TNFAIP6	6.97	-6.07	7.39
Hs.701982	interleukin 1 receptor, type I	IL1R1	2.64	-2.21	4.00
Hs.525572	bradykinin receptor B1	BDKRB1	2.48	-2.51	2.99
Hs.534847	complement component 4A (Rodgers blood group)	C4A	2.16	-2.03	2.66
Hs.196384	prostaglandin-endoperoxide synthase 2	PTGS2	2.07	-2.96	3.05
Hs.81791	tumor necrosis factor receptor superfamily member 11b	TNFRSF11B	2.00	-2.77	3.65
Hs.203717	fibronectin 1	FN1	2.31	-2.57	3.84
Hs.654458	interleukin 6 (interferon, beta 2)	IL6	5.29	-2.27	6.10
**Metabolic process**					
Hs.387367	cytochrome P450, family 39, subfamily A, polypeptide 1	CYP39A1	11.32	-7.58	12.30
Hs.460260	aldo-keto reductase family 1, member C1	AKR1C1	9.85	-3.45	8.47
Hs.567256	aldo-keto reductase family 1, member C2	AKR1C2	3.10	-2.37	2.90
Hs.116724	aldo-keto reductase family 1, member B10	AKR1B10	3.03	-2.10	2.83
Hs.78183	aldo-keto reductase family 1, member C3	AKR1C3	2.65	-2.07	2.30
Hs.419240	solute carrier family 2, member 1	SLC2A14	2.60	-2.91	3.22
Hs.419240	solute carrier family 2, member 3	SLC2A3	2.46	-2.17	3.91
Hs.572518	UDP-glucose dehydrogenase	UGDH	2.00	-2.77	3.14
**Protein amino acid dephosphorylation**					
Hs.160871	protein tyrosine phosphatase, receptor type, O	PTPRO	3.25	-2.31	4.20
Hs.43666	protein tyrosine phosphatase type IVA, member 3	PTP4A3	2.46	-2.83	4.66
Hs.497822	dual specificity phosphatase 10	DUSP10	2.14	-3.15	3.02
**Other up-regulated gene expression**					
Hs.459265	interferon stimulated exonuclease gene 20 kDa	ISG20	9.19	-4.57	8.10
Hs.118633	2'-5'-oligoadenylate synthetase-like	OASL	8.00	-4.82	6.26
Hs.144873	BCL2/adenovirus E1B 19 kDa interacting protein 3	BNIP3	2.12	-2.35	4.19

### Major functional categories of downregulated genes in response to hypoxia by HIF-1alpha

Among the 28 genes that showed more than 2.0-fold decreased expression, were genes in pathways such as protein amino acid phosphorylation, growth factors/cytokines, cell adhesion/motility, transcription, transport and others(Table 2). Just like the categories of genes upregulated by HIF-1alpha, the largest category of genes that were downregulated were genes that encode transport factors (including two members of SLC gene family: SLC16A14 and SLC35F3). The genes encoding growth factors/cytokines included SOCS2 and IGFBP3, which are in the same gene families with SOCS1 and IGFBP5, respectively. The expression of IGFBP3, ZNF569, SOCS2, SIRPa and XRCC4 genes were analyzed by real-time PCR to validate. The results also showed a similar trend of regulation as the microarray data (Figure 2B).

**Table 2:** 28 genes downregulated by HIF-1alpha more than 2.0-fold in three pairwise comparisons

UniGeneID	Gene name	Gene Symbol	Fold change(ratio ≥ 2)		
			
			Ad5-HIF-1alpha/Ad5	Ad5-siHIF-1alpha/Ad5	Hypoxia /normoxia
**Transport**					
Hs.666728	Na+/H+ exchanger domain containing 1	NHEDC1	-27.86	9.86	-12.33
Hs.666367	potassium voltage-gated channel, Shal-related subfamily, member 3	KCND3	-16.00	6.13	-11.82
Hs.581021	signal-regulatory protein alpha	SIRPa	-4.93	3.10	-3.72
Hs.504317	solute carrier family 16, member 14 (monocarboxylic acid transporter 14)	SLC16A14	-4.59	2.46	-4.30
Hs.118695	potassium voltage-gated channel, subfamily G, member 1	KCNG1	-2.13	2.35	-3.17
Hs.158748	solute carrier family 35, member F3	SLC35F3	-2.06	2.76	-2.55
Hs.443625	collagen, type III, alpha 1	COL3A1	-2.29	2.16	-3.78
**Transcription**					
Hs.458406	undifferentiated embryonic cell transcription factor 1	KCNG1	-36.76	12.17	-45.69
Hs.511848	zinc finger protein 569	ZNF569	-12.13	7.61	-15.33
Hs.412196	intraflagellar transport 57 homolog	IFT57	-8.58	4.38	-7.36
Hs.533977	thioredoxin interacting protein	TXNIP	-5.28	3.10	-5.01
Hs.4779	GATA zinc finger domain containing 2B	GATAD2B	-3.48	2.31	-6.30
Hs.9521	zinc finger protein 92	ZNF92	-2.83	2.09	-3.19
Hs.490273	cAMP responsive element binding protein3-like 2	CREB3L2	-2.07	2.00	-3.12
Hs.524248	zinc finger protein 362	ZNF362	-2.00	2.67	-4.78
**Growth factors/cytokines**					
Hs.485572	suppressor of cytokine signaling 2	SOCS2	-6.06	3.06	-7.12
Hs.450230	insulin-like growth factor binding protein 3	IGFBP3	-4.02	2.17	-5.73
Hs.8867	cysteine-rich, angiogenic inducer, 61	CYR61	-3.03	2.18	-3.77
Hs.289008	nuclear undecaprenyl pyrophosphate- synthase 1 homolog	NUS1	-2.83	2.13	-4.01
Hs.699288	neural precursor cell expressed, developmentally down-regulated 9	NEDD9	-2.64	2.26	-2.57
**Protein amino acid phosphorylation**					
Hs.370503	FYN binding protein (FYB-120/130)	FYB	-6.06	3.97	-4.71
Hs.460355	protein kinase C, beta 1	PRKCB1	-3.25	2.56	-4.30
Hs.390729	v-erb-a erythroblastic leukemia viral oncogene homolog 4	ERBB4	-2.46	2.11	-3.89
Hs.654491	receptor tyrosine kinase-like orphan receptor 1	ROR1	-2.47	2.32	-4.56
Hs.653377	insulin-like growth factor 1 receptor	IGF1R	-2.00	2.89	-3.11
**Other down-regulated gene expression**					
Hs.606356	pleckstrin homology domain interacting protein	PHIP	-17.15	4.76	-10.03
Hs.567359	X-ray repair complementing defective repair in Chinese hamster cells 4	XRCC4	-8.00	6.21	-5.69
Hs.502182	brain-derived neurotrophic factor	BDNF	-2.30	2.14	-2.18

### Effects of HIF-1alpha and hypoxia on SOCS1, IGFBP5, IL-6 and STAT3 protein expression in NCI-H446 cells

**Figure 3:**
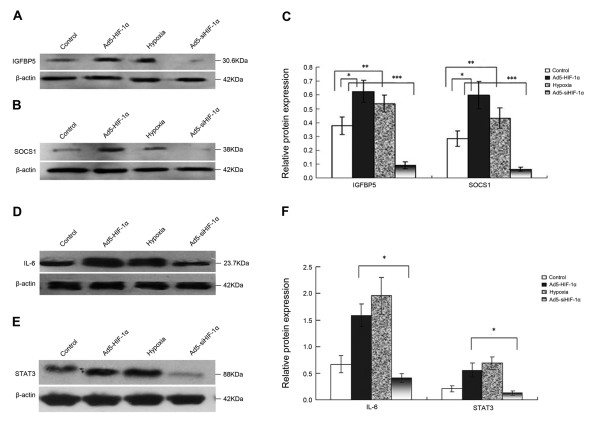
**Western blot analysis of regulation of protein expression by HIF-1alpha in NCI-H446 cells**. According to different treatments, all the cells were divided into four groups: control group (the cells cultured under normoxic conditions of 20% O2), Ad5-HIF-1alpha transfection group, hypoxia group (the cells cultured under normoxic conditions of 1% O2) and Ad5-siHIF-1alpha transfection group (after transfection, the cells were cultured under normoxic conditions of 1% O2). **(A) **Western blot analysis for IGFBP5 protein expressed by the cells of four groups. **(B) **Western blot analysis for SOCS1 protein expressed by the cells of four groups. **(C) **Densitometric analysis of the IGFBP5 and SOCS1 bands compared to the corresponding β-actin bands (*p < 0.05 expression of IGFBP5 or SOCS1 protein in Ad5-HIF-1alpha group vs. control group; ** p < 0.05 expression of IGFBP5 or SOCS1 protein in hypoxia group vs. control group; *** p < 0.05 expression of IGFBP5 or SOCS1 protein in Ad5-siHIF-1alpha group vs. control group). **(D) **Western blot analysis for IL-6 protein expressed by the cells of four groups. **(E) **Western blot analysis for STAT3 protein expressed by the cells of four groups. **(F) **Densitometric analysis of the IL-6 and STAT3 bands compared to the corresponding β-actin bands (*p < 0.05 expression of IL-6 or STAT3 protein in Ad5-HIF-1alpha group vs. Ad5-siHIF-1alpha group group.)

It is well known that regulation at the mRNA level does not always predict regulation at the protein level. Hence, we investigated the changes in the expression levels of SOCS1 and IGFBP5 proteins by Western blot analysis. SOCS1 and IGFBP5, which were upregulated by HIF-1alpha at the mRNA level, are closely related with biological properties of tumor (as we will describe in the discussion). Result of the Western blot analysis (Figure 3A, B, C) showed that the protein levels of SOCS1 and IGFBP5 were significantly upregulated after transfection with HIF-1alpha or after culturing cells in the hypoxic environment but were significantly downregulated after transfection with siHIF-1alpha. Besides two factors above-mentioned, inflammatory factor IL-6 and downstream signal transducer STAT3 were also upregulated at the protein level in Ad5-HIF-1alpha group and hypoxia group especially in hypoxia group but downregulated in Ad5-siHIF-1alpha group (Figure 3D, E, F).

### Effect on cell growth and apoptosis by HIF-1alpha and SOCS1

**Figure 4:**
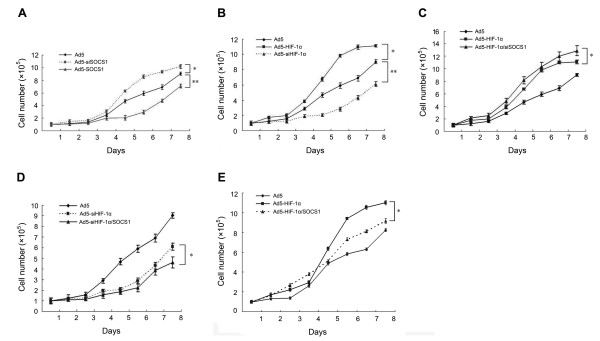
**Effect of HIF-1alpha and SOCS1 on cell growth was measured by cell counting**. **(A) **After transfection with Ad5-SOCS1, the growth of cells was slowed but promoted after transfection with Ad5-siSOCS1(*p < 0.05 Ad5-siSOCS1 group vs. Ad5 group; **p < 0.01 Ad5-SOCS1 group vs. Ad5 group) **(B) **After transfection with Ad5- HIF-1alpha, the growth of cells was promoted but slowed after transfection with Ad5- siHIF-1alpha (*p < 0.01 Ad5-HIF-1alpha group vs. Ad5 group; **p < 0.01 Ad5-si HIF-1alpha group vs. Ad5 group). **(C) **In the Ad5-HIF-1alpha group, the growth of cells was promoted after blockade of SOCS1 by Ad5-siSOCS1 (*p < 0.01 Ad5- HIF-1alpha/siSOCS1 group vs. Ad5-HIF-1alpha group). **(D) **In the Ad5-si HIF-1alpha group, the growth of cells was slowed after co-transfection with SOCS1 (*p < 0.05 Ad5-siHIF-1alpha/SOCS1 group vs. Ad5-siHIF-1alpha group). **(E) **In the Ad5-HIF-1alpha group, the growth of cells was slowed from day 5 to day 8 as the growth curve moved right after co-transfection with SOCS1 (*p < 0.05 Ad5-HIF-1alpha group vs. Ad5-HIF-1alpha/SOCS1 group).

**Figure 5:**
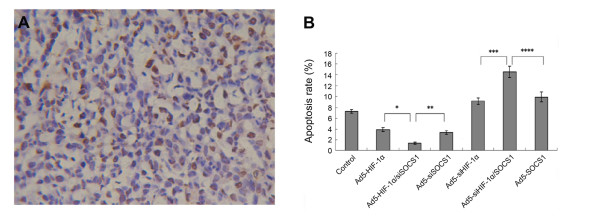
**We used the tunel stain to investigate the effect of HIF-1alpha and SOCS1 on cell apoptosis and the apoptosis rate was calculated in all the experimental groups**. **(A) **The background was clear and the apoptotic NCI-H446 nucleuses were stained yellow and normal nucleuses were stain blue(tunel stain × 400) **(B) **The effect of HIF-1alpha and SOCS1 on apoptosis of SCLC cells after transfection for 8 d (*p < 0.05 Ad5-HIF-1alpha/siSOCS1 group vs. Ad5-HIF-1alpha group; **p < 0.05 Ad5-HIF-1alpha/siSOCS1 group vs. Ad5-siSOCS1 group; ***p < 0.01 Ad5-si HIF-1alpha/SOCS1 group vs. Ad5-siHIF-1alpha group; ****p < 0.05 Ad5-si HIF-1alpha/SOCS1 group vs. Ad5-SOCS1 group).

Transfection with Ad5-HIF-1alpha increased the growth rate of NCI-H446 cells, and the growth rate of NCI-H446 cells decreased after transfection with Ad5-si HIF-1alpha; however, the effects of SOCS1 were the opposite (Figure 4A, B). The growth curve of the co-transfection group (Figure 4C, D) confirmed the above-mentioned result. In Ad5-HIF-1alpha/SOCS1 group we could see that in exponential phase (from 5-8 days) the growth rate of cells also decreased comparing to Ad5-HIF-1alpha group (Figure 4E). In the assay of tunel stain the apoptotic cells were stained yellow for counting (Figure 5A). Analysis of the apoptosis rate demonstrated that HIF-1alpha inhibited apoptosis of NCI-H446 cells, but SOCS1 induced apoptosis (Figure 5B). Analysis of the co-transfection groups, such as the Ad5-HIF-1alpha/siSOCS1 group and Ad5-siHIF-1alpha/SOCS1 group, displayed an inter-antagonism between HIF-1alpha and SOCS1 in regulating apoptosis of NCI-H446 cells even though HIF-1alpha upregulated the expression of SOCS1 at both the mRNA and protein levels (Figure 5B).

## Discussion

Tissue hypoxia is critical in the process of tumor formation. Activation of HIF-1 alpha, an important transcription factor that is expressed in response to hypoxia, is a common feature of tumors and is generally more pronounced in aggressive solid tumors such as SCLC and can even be an independent predictor of prognosis in certain types of cancer [9,10]. To characterize the molecular mechanisms involved in the carcinogenesis, progression and prognosis of SCLC which are regulated by HIF-1 alpha and identify genes to be applied as novel diagnostic markers or for development of gene targeted therapy, we applied cDNA microarray profile analysis and integrated the results of gene expression profiles of the hypoxia, HIF-1 alpha and siHIF-1 alpha groups. In this way, we could eliminate the effects on gene expression by others factors involving the hypoxic microenvironment and stringently screened out the genes regulated by HIF-1alpha. From the results of three pairwise comparisons we found that both upregulated and downregulated genes were all associated with these categories: transport, growth factors/cytokines, transcription and protein amino acid phosphorylation.

Angiogenesis is essential for progression, invasion and metastasis of SCLC[11]. As a specific target of most tumors VEGF is a target gene of HIF-1 alpha and plays a main role in control of angiogenesis both in physiological and pathological situations, including tumor development and progression. It is mitogenic and angiogenic for endothelial cells, and it can also increase vascular permeability [12]. Identical with previous study [13] our study also found that VEGF-A was upregulated by HIF-1 alpha more than 6-fold in SCLC. But besides VEGF-A, there are several other genes associated with angiogenesis such as PDGFC, PLA2G4A, HMOX1, HMGA2 were upregulated by HIF-1 alpha. These genes were not reported in others literatures and therefore we think the upregulation of these genes may be specific to the angiogenesis of SCLC when responding to HIF-1 alpha or hypoxia.

Some genes had been reported to be found with differential expression in SCLC through microarray analysis. Amplification and overexpression of the MYC family of oncogenes such as MYC (c-Myc), MYCN (N-Myc) and MYCL1 (L-Myc) occured in SCLCs [14] and was common in chemo-refractory disease[15]. In our study not MYC family but SLC family such as SLC6A2 and SLC9A2 were upregulated by HIF-1 alpha. Some genes as TAF5L, TFCP2L4, PHF20, LMO4, TCF20 and RFX2 that were known to have transcription factor activities express highly in SCLC[16] but the genes that were upregulated by HIF-1 alpha are TRIM22, IRF9, MYOCD, ZNF277 and CREM from our study. Previous study also reported that the high expression of BAI3, D4S234E, DCX, DPYSL5 and GKAP1 which were related to signal transduction were found in SCLC [16,17]. In our study signal transduction factor IRS4 and GPER1 were upregulated by HIF-1 alpha more than 6.0-fold. As for IRS4 some researchers have found that it plays an important role in proliferation/differentiation of tumors and exerts its actions through ERK and p70S6K activation in a ras/raf/MEK1/2 and PI3K/Akt independent manner and in a PKC-dependent way [18]. The GPER1 gene (also known as GPR30) represents an alternative estrogen-responsive receptor, which is highly expressed in tumors where estrogen and progesterone receptors are downregulated and in high-risk tumor patients with lower survival rates[19]. GPER1 is also an important mediator of some single transduction pathways contributing to promote proliferation, metastasis and aggressive behaviors of tumors that are induced by endogenous estrogens, including drugs like hydroxytamoxifen and atrazine or the environmental pollutant cadmium [20-22].

A novel finding different from previous study is that some genes encoding inflammatory response cytokines were upregulated. This maybe provides a broad molecular-biological basis for the inflammatory effect of SCLC. The highest upregulation are TNFAIP6 and IL6. TNFAIP6 can inhibit osteoblastic differentiation of human mesenchymal stem cells [23]. From this, we might conclude that this is one potential mechanism of SCLC-mediated osteoclasia through upregulation of TNFAIP6 gene expression by HIF-1 alpha. High expression of IL6 also is associated with malignant tumors and deep vein thrombosis disease [24]. This helps to explain why a hypercoagulable state is usually associated with SCLC and what causes the thrombosis. Another novel finding is that with the genes in the same family HIF-1 alpha upregulates the expression of SOCS1 but downregulates the expression of SOCS2, upregulates the expression of IGFBP5 but downregulates the expression of IGFBP3. Clinical research has shown that SOCS2 is an independent predictor for good prognosis, negative lymph nodes and has increased expression in less malignant tumors [25]. From this the downregulation of SOCS2 by HIF-1 alpha maybe worsen the prognosis of SCLC. Besides these increased IGFBP-5 mRNA levels have been proved to be associated with a poor outcome for the patients who have positive lymph nodes[26] and high circulating concentrations of IGFBP-3 is associated with a lower cancer risk from clinical trail[27]. Thus upregulation of IGFBP5 and downregulation of IGFBP3 by hypoxia or HIF-1 alpha cannot be considered as good predictors of prognosis. As for SOCS1 some scholars have demonstrated that SOCS1 plays an important role in degrading IFN resistance of neuroendocrine tumor cells through negative regulation of Jak/STAT signaling pathway[28]. Our study demonstrates that SOCS1 potentially induces the apoptosis and suppresses the growth of NCI-H446 cells and therefore we thought the upregulation of SOCS1 may be a good predictor for the prognosis of SCLC. As an upstream regulatory factor that plays a contrary effect on the apoptosis and growth of SCLC cells, through which mechanism HIF-1 alpha upregulates the expression of.SOCS1, is a new problem to investigate. Previous study had demonstrated that the activation of STAT3 mediated by IL-6 could upregulate the expression of SOCS1 at mRNA and protein level [29]. From our study we can see that the expression of STAT3 and IL-6 are both upregulated at protein level. So we can primarily conclude that HIF-1 alpha can upregulate the expression of SOCS1 through mediation of STAT3 and IL-6. As for the signal transduction pathway involving in this pocess we will carry out some resrarch work in the future.

Taken together, our data provide novel insights into the composition and function of differential gene expression regulated by HIF-1alpha in SCLC NCI-H446 cells. Improving the survival rate of patients with SCLC requires a better understanding of the function of genes associated with tumorigenesis and the subsequent development of novel gene therapeutic strategies including gene targeted therapy. Several categories of targeted genes have been introduced into clinical trials of SCLC [30] such as matrix metalloproteinase inhibitors, anti-angiogenic agents receptor and tyrosine kinase inhibitors. From our study it seems that regardless of upregulation or downregulation of these functional genes, the trend of the tumor is to deteriorate due to the abnormal expression of genes mediated by HIF-1alpha. Our work aims to find more novel functional genes whose expression is mediated by HIF-1alpha to support the development of new therapeutic targets for gene targeted therapy of SCLC.

## Conflicting interests

The authors declare that they have no competing interests.

## Authors' contributions

JW carried out the experimental studies, participated in the literature research and drafted the manuscript. JBM participated in the experimental studies. GS participated in the sequence alignment, the design of the study and performed the data analysis. JM conceived of the study, and participated in its design and helped to draft the manuscript. All authors read and approved the final manuscript.
